# Comparison between compressed sensing and segmented cine cardiac magnetic resonance: a meta-analysis

**DOI:** 10.1186/s12872-023-03426-1

**Published:** 2023-09-21

**Authors:** Jason Craft, Yulee Li, Niloofar Fouladi Nashta, Jonathan Weber

**Affiliations:** 1 DeMatteis Cardiovascular Institute, St. Francis Hospital & Heart Center, 100 Port Washington Blvd, Roslyn, NY 11576 USA; 2 Sol Price School of Public Policy and Leonard D. Schaeffer Center for Health Policy and Economics, University of Southern California, Los Angeles, CA USA

**Keywords:** Review article, Compressed sensing, Real-time cine, Cine MRI

## Abstract

**Purpose:**

Highly accelerated compressed sensing cine has allowed for quantification of ventricular function in a single breath hold. However, compared to segmented breath hold techniques, there may be underestimation or overestimation of LV volumes. Furthermore, a heterogeneous sample of techniques have been used in volunteers and patients for pre-clinical and clinical use. This can complicate individual comparisons where small, but statistically significant differences exist in left ventricular morphological and/or functional parameters. This meta-analysis aims to provide a comparison of conventional cine versus compressed sensing based reconstruction techniques in patients and volunteers.

**Methods:**

Two investigators performed systematic searches for eligible studies using PubMed/MEDLINE and Web of Science to identify studies published 1/1/2010-3/1/2021. Ultimately, 15 studies were included for comparison between compressed sensing cine and conventional imaging.

**Results:**

Compared to conventional cine, there were small, statistically significant overestimation of LV mass, underestimation of stroke volume and LV end diastolic volume (mean difference 2.65 g [CL 0.57–4.73], 2.52 mL [CL 0.73–4.31], and 2.39 mL [CL 0.07–4.70], respectively). Attenuated differences persisted across studies using prospective gating (underestimated stroke volume) and non-prospective gating (underestimation of stroke volume, overestimation of mass). There were no significant differences in LV volumes or LV mass with high or low acceleration subgroups in reference to conventional cine except slight underestimation of ejection fraction among high acceleration studies. Reduction in breath hold acquisition time ranged from 33 to 64%, while reduction in total scan duration ranged from 43 to 97%.

**Conclusion:**

LV volume and mass assessment using compressed sensing CMR is accurate compared to conventional parallel imaging cine.

**Supplementary Information:**

The online version contains supplementary material available at 10.1186/s12872-023-03426-1.

## Introduction

Cardiac MRI (CMR) is the gold standard for quantification of left ventricular volume and function [1]. However, balanced steady-state free precession (bSSFP) segmented cardiac cine is prone to corruption by cardiac and respiratory motion. Conventional parallel imaging techniques have led to a shorter breath hold duration at an expense of signal to noise, but do not address the limitations of the segmented technique. Real-time cine imaging with conventional parallel imaging is not sufficient in many instances, sacrificing spatial, temporal resolution, and overall image fidelity. Over the last few years, compressed sensing (CS) cardiac cine entered the investigational phase and now is commercially available. Therefore, CS cine has served to meet a clinical need, giving clinicians the ability to image critically ill patients with limited cardiorespiratory reserve. Despite the overall high quality of CS cine, some previous studies have suggested statistically significant difference trends in left and/or right ventricular volumetric data, while others have not [2–16]. Historically, several sampling algorithms and reconstruction techniques have been explored. However, only a select few have been established and been made available for on-scanner reconstruction, and thus clinical use. Therefore, the purpose of this meta-analysis is to summarize the expected differences in left ventricular structure and function parameters using clinically feasible CS cine versus the reference breath hold cine.

## Methods

### Search Strategy

This study followed the recommendations of the preferred reporting items for systematic reviews and meta-analyses statement (PRISMA). Two investigators (NFN and JC) performed systematic searches for eligible studies using PubMed/MEDLINE and Web of Science to identify studies published between 1/1/2010 and 3/1/2021. Search terms included “compressed sense cine,” “compressed sensing cine,” and “compressed sensing cine NOT Non-iterative reconstructions.”

### Study selection

First, titles and abstracts found by searches were assessed for eligibility by one author and verified by another (NFN and JW). After consensus was reached, full texts of preliminarily eligible studies were extracted and independently assessed by two investigators (JC and JW) against criteria for final inclusion. Study quality was independently evaluated by two authors (JC and JW) using the COSMIN Risk of Bias tool to assess the quality of studies on reliability and measurement error of outcome measurement instrument scale [17].

**Table 1:** Study-specific differences in left ventricular ejection fraction

Study name	Sample Size	Standardized difference	95% CL	p value		Mean difference, (%)	95% CL	p value	Weight
Allen, et al. Int J Cardiovasc Imaging 2016 [2]	29	0.08	(-0.66, 0.82)	0.840		0.70	(-6.11, 7.51)	0.840	1.3
Ma et al. Clinical Radiology 2019 [3]	33	-0.32	(-0.67, 0.03)	0.073		-0.40	(-0.83, 0.03)	0.066	5.6
Kido et al. JCMR 2021 [4]	65	-0.20	(-0.45, 0.04)	0.104		-1.10	(-2.41, 0.21)	0.101	11.4
Kido et al. JCMR 2016 [5]	81	-0.03	(-0.47, 0.41)	0.903		-0.40	(-6.82, 6.02)	0.903	3.6
Goebel et al. JMRI 2016 [6]	16	-0.18	(-0.68, 0.31)	0.470		-2.00	(-7.38, 3.38)	0.466	2.8
Goebel et al. Eur Radiology 2016 [7]	26	-0.38	(-1.16, 0.39)	0.336		-4.00	(-12.07, 4.07)	0.332	1.1
Goebel et al. Acta Radiology 2017 [8]	20	-0.37	(-0.83, 0.08)	0.106		-3.80	(-8.25, 0.65)	0.094	3.3
Allen, et al. Eur Radiology 2018 [9]	27	0.07	(-0.7, 0.84)	0.863		0.90	(-9.34, 11.14)	0.863	1.2
Sudarski et al. Radiology 2016, [10] Patients	50	-0.29	(-0.57, -0.01)	0.046		-1.00	(-1.96, -0.04)	0.042	8.6
Sudarski et al. Radiology 2016, [10] Ctrl subjects	10	-0.17	(-0.8, 0.45)	0.592		-0.80	(-3.7, 2.1)	0.589	1.8
Naresh et al. Pediatric Radiology 2020 [11]	28	0.07	(-0.3, 0.44)	0.704		2.00	(-8.32, 12.32)	0.704	5.0
Kocaoglu et al. J Cardiovasc Magn Reson 2020 [12]	26	0.07	(-0.31, 0.46)	0.716		0.20	(-0.88, 1.28)	0.716	4.6
Wang et al. SS CS Cardiovasc Diagn Ther 2020 [13]	38	-0.04	(-0.36, 0.28)	0.800		-0.40	(-3.5, 2.7)	0.800	6.8
Wang et al. 2-shot SS Cardiovasc Diagn Ther 2020 [13]	38	0.02	(-0.3, 0.33)	0.923		0.10	(-1.93, 2.13)	0.923	6.8
Wang et al. Int J Cardiovasc Imaging 2020 [14]	121	0.00	(-0.18, 0.18)	1.000		0.00	(-0.06, 0.06)	1.000	21.6
Lin et al. J Magn Reson Imaging 2017 [15]	50	0.16	(-0.12, 0.44)	0.261		0.40	(-0.29, 1.09)	0.258	8.8
Vincenti et al. JACC Cardiovasc Imaging 2014 [16]	33	-0.30	(-0.65, 0.05)	0.089		-1.30	(-2.77, 0.17)	0.082	5.6
Pooled (random effects) model		-0.09	(-0.17, -0.01)	0.038		-0.20	(-0.47, 0.06)	0.134	

Differences in LVEF between compressed-sensing and control sequences are presented as standardized difference and mean difference with effects pooled using a random effects model

#### Inclusion criteria

Studies used for the purpose of this meta-analysis compared reference segmented bSSFP vs. CS cine. Numerous approaches to CS reconstruction have been employed to accelerate cine imaging in pre-clinical and clinical CMR. To ensure relevance and compatibility with everyday clinical practice, only Cartesian data sampling methods with on-scanner reconstruction were considered. These typically included a pseudo-random sampling strategy in k-t space, an iterative SENSE (sensitivity encoding)-like reconstruction algorithm, and a CS sparsity constraint along the k-space phase encoding direction and the temporal space [18–20].

#### Exclusion criteria

Studies using radial, spiral, or other alternative k-space trajectories and reconstruction algorithms not available for clinical use because of lengthy and/or offline reconstruction were excluded. This removes some degree of heterogeneity across studies.

### Statistical analysis

Variables extracted for analysis from full texts included CS cine and reference left ventricular end-systolic (LVESV) and end-diastolic volumes (LVEDV), stroke volume (LVSV), ejection fraction (LVEF), and mass (LVM). Data formats extracted included raw group means/standard deviation, or mean differences/mean difference standard deviation. Volumetric variables and mass of CS cine sequences were compared with control sequences. Comparisons were displayed as CS measurement minus reference measurement whereas underestimation and overestimation by CS sequences is displayed as negative or positive differences, respectively. Mean differences and standardized differences were presented and pooled analyses were performed using random-effect models. *I*^*2*^ tests for heterogeneity were performed for each volumetric variable and mass whereby *I*^*2*^ values of > 50% were considered substantial according to the Cochrane handbook. Publication bias was assessed through the use of funnel plots with the trim-and-fill method along with Egger’s test and Kendall’s Tau. Two-sided p values < 0.05 were considered statistically significant. All analyses were performed using Comprehensive Meta Analysis software version 3 (Biostat Inc., Englewood, NJ).

## Results

**Fig. 1:**
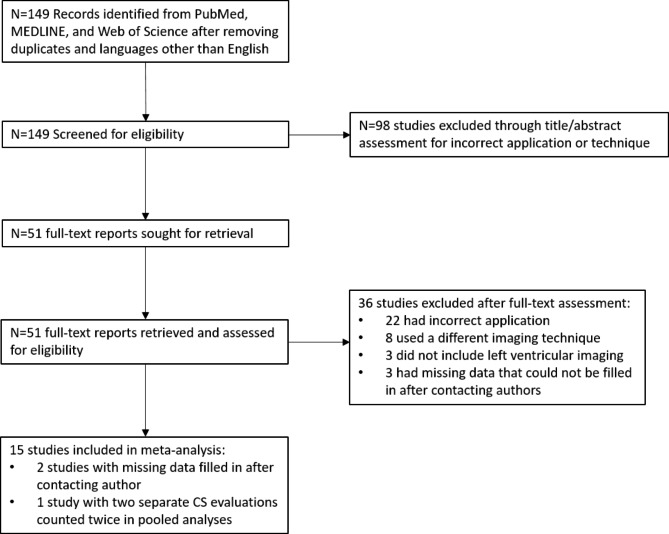
**PRISMA flow-chart.** There were 15 studies included in the meta-analysis after screening 149 records from PubMed, MEDLINE and Web of Science. There were 2 studies whose missing data was filled in after contacting authors and 1 study that contributed two sets of compressed sensing comparisons and was included twice in pooled analyses

There were 149 studies found in initial searches. After screening titles and abstracts for eligibility, 51 full-text studies were extracted and assessed. There were 15 studies which met study inclusion criteria. 1 study included two entirely separate CS evaluations and was included twice in pooled analyses (Fig. 1). There were 5 studies with missing data required for comparison among which 2 study were included after contacting co-authors for missing information. One study presented healthy control subjects and disease subjects separately.

### Study specific acquisition parameters

CS cine acquisition parameters and details for each study are displayed in tables S1 and S2 of the online supplement. All 15 studies used an online CS cine prototype developed with a CS technique combined with SENSE. These prototypes featured a pseudo-random sampling trajectory in k-t space and an iterative reconstruction algorithm. Among them, 12 were implemented on Siemens MRI scanners and 3 on Philips MRI scanners. The median sample size was 33 (range 16–121). Seven studies used CS prospective gating (segmented or single shot), six studies used ungated CS real-time acquisitions (no EKG) or retrospective (segmented) gating; one study used both retrospective/prospective gating; and one study was unknown. There were 9 studies which included papillary muscle in volumetric analyses while 1 excluded and 5 were not reported. Acceleration factors ranged from 2.5 to 13. Notably, 8 studies were performed on 1.5T MRI systems with the remaining performed on 3T systems. Compared with the control sequences, CS cine resulted in a reduction in overall scan time of between 43 and 97%, with a reduction of breath hold duration (when applicable) ranging from 33 to 64%.

### Global differences in LV mass, volumes, and analysis of gating

**Fig. 2:**
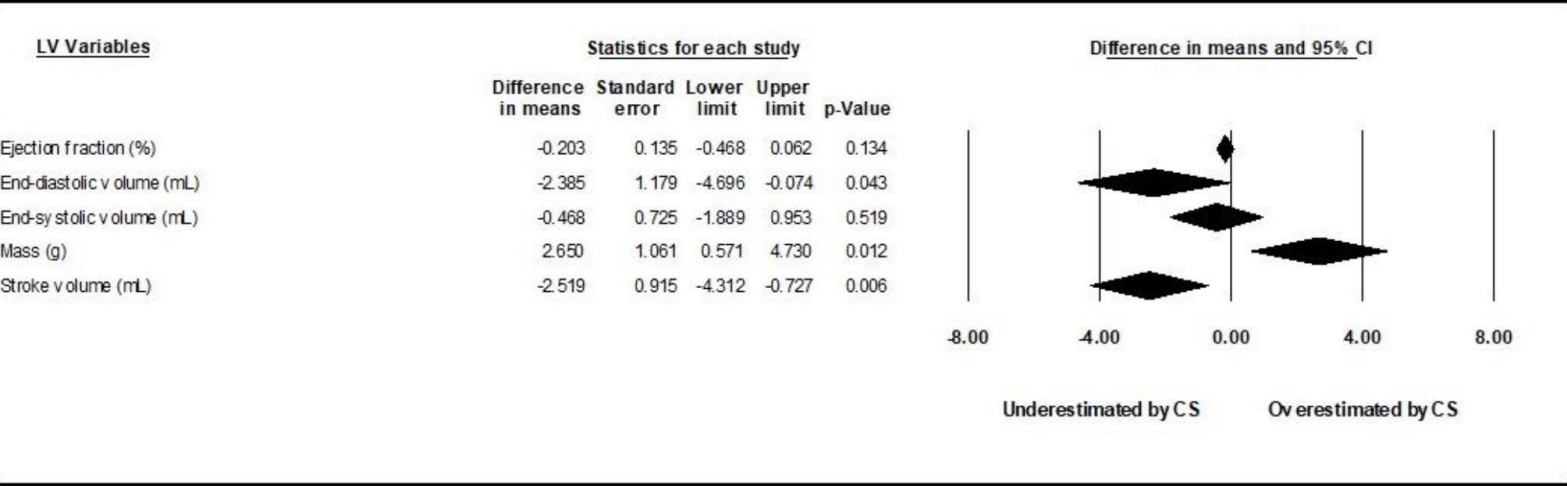
**Pooled results, all studies.** Comparison between compressed-sensing and control sequences. Pooled comparisons of left ventricular parameters between compressed-sensing and control sequences among included studies demonstrated small, statistically significant differences underestimations of LVEDV and LVSV, as well as slight overestimation of LV mass compared with the reference group (*p* ≤ 0.05)

**Fig. 3:**
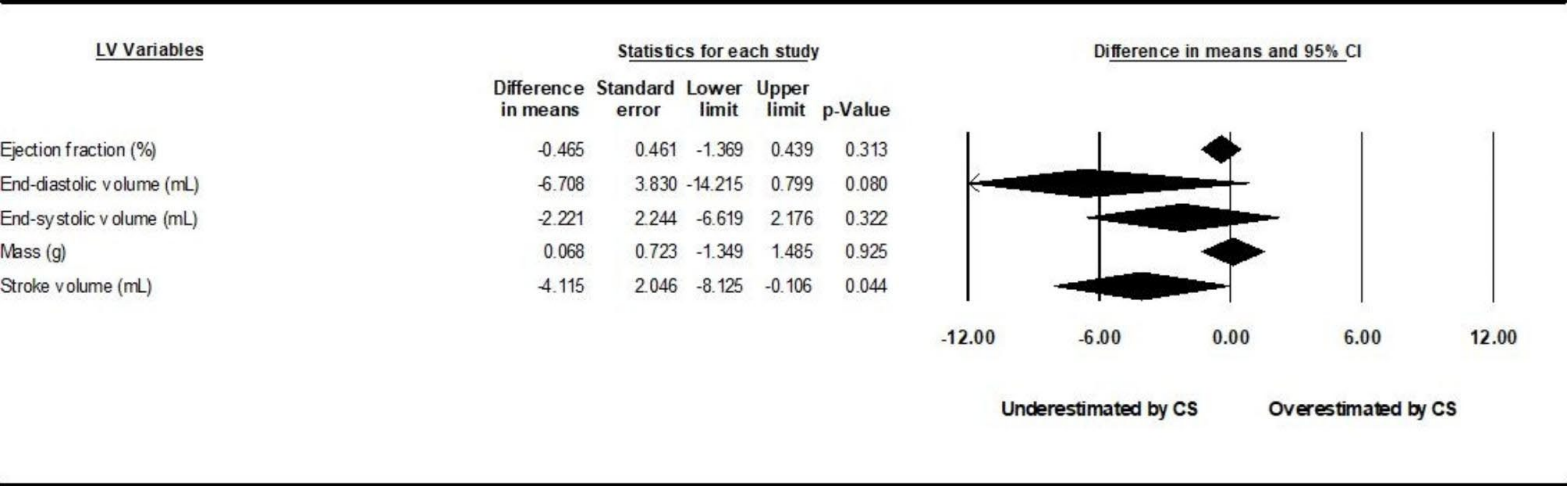
**Subgroup analysis results, prospective gating.** Comparison of sequences stratified by gating method. Left ventricular parameter comparisons stratified by prospective gating yielded similar but attenuated underestimation of stroke volume

**Fig. 4:**
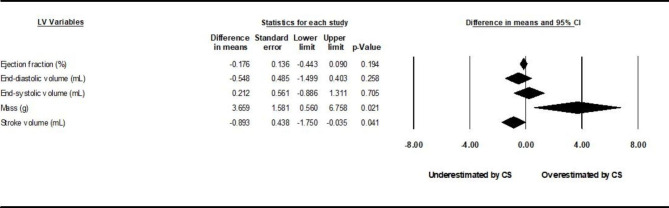
**Subgroup analysis results, non-prospective gating or no gating.** Comparison of sequences stratified by gating method. Ungated/retrospective gating demonstrated slight overestimation of LV mass and underestimation of LVSV compared with reference

Pooled mean differences between CS cine sequences and control sequences are reported in Fig. 2 for LV volumes, LVEF, and LVM using random-effects models. Underestimation by CS cine sequences of LVEDV (by 2.4 mL) and LVSV (by 2.5 mL), and slight overestimation of LVM (by 2.7 g) were found to be statistically significant (p < 0.05). Study-specific comparisons including mean differences and standardized differences are reported in Tables 1, 2, 3, 4 and 5. The *I*^*2*^ values were in the range of 3–30%, indicating likely non-significant heterogeneity. Risk of bias evaluated using the COSMIN checklist yielded very consistent results between raters. Risk of bias in individual studies appears low with all bias metrics ranked as “very good” or “adequate” by both raters. Risk of publication bias evaluated through the use of funnel plots and the trim-and-fill method yielded bias-corrected results very similar to original standard differences. Interdependence of variance and effect size as well as asymmetry of the funnel plots were also not found through the use of Kendall’s tau and Egger’s test, respectively (table S3 of the online supplement). Subgroup analyses among studies using prospective vs. non prospective gating are reported in Figs. 3 and 4. Again, there is redemonstration of statistically significant but attenuated minimal differences in LVSV for prospectively gated studies, and LVSV and LVM for non-prospective or non-gated studies.

**Table 2:** Study-specific differences in left ventricular end-diastolic volume

Study name	Sample Size	Standardized difference	95% CL	p value		Mean difference, mL	95% CL	p value	Weight
Allen, et al. Int J Cardiovasc Imaging 2016 [2]	29	0.13	(-0.61, 0.88)	0.722		6.80	(-30.68, 44.28)	0.722	3.2
Ma et al. Clinical Radiology 2019 [3]	33	-0.06	(-0.4, 0.28)	0.726		-0.23	(-1.51, 1.05)	0.725	6.8
Kido et al. JCMR 2021 [4]	65	-0.04	(-0.28, 0.21)	0.771		-0.30	(-2.32, 1.72)	0.771	8.0
Kido et al. JCMR 2016 [5]	81	-0.03	(-0.47, 0.41)	0.900		-1.20	(-19.94, 17.54)	0.900	5.7
Goebel et al. JMRI 2016 [6]	16	-0.13	(-0.62, 0.36)	0.611		-5.60	(-27.12, 15.92)	0.610	5.1
Goebel et al. Eur Radiology 2016 [7]	26	-0.15	(-0.92, 0.62)	0.706		-6.00	(-37.18, 25.18)	0.706	3.0
Goebel et al. Acta Radiology 2017 [8]	20	0.32	(-0.13, 0.77)	0.166		5.90	(-2.25, 14.05)	0.156	5.5
Allen, et al. Eur Radiology 2018 [9]	27	-0.18	(-0.95, 0.59)	0.640		-9.40	(-48.7, 29.9)	0.639	3.0
Sudarski et al. Radiology 2016, [10] Patients	50	-0.22	(-0.5, 0.06)	0.130		-1.90	(-4.33, 0.53)	0.125	7.5
Sudarski et al. Radiology 2016, [10] Ctrl subjects	10	-0.07	(-0.69, 0.55)	0.823		-0.60	(-5.85, 4.65)	0.823	4.0
Naresh et al. Pediatric Radiology 2020 [11]	28	-0.07	(-0.44, 0.3)	0.706		-2.00	(-12.36, 8.36)	0.705	6.4
Kocaoglu et al. J Cardiovasc Magn Reson 2020 [12]	26	-0.29	(-0.68, 0.1)	0.148		-2.00	(-4.65, 0.65)	0.139	6.2
Wang et al. SS CS Cardiovasc Diagn Ther 2020 [13]	38	0.07	(-0.25, 0.38)	0.686		1.00	(-3.84, 5.84)	0.685	7.1
Wang et al. 2-shot SS Cardiovasc Diagn Ther 2020 [13]	38	0.06	(-0.26, 0.38)	0.706		0.70	(-2.93, 4.33)	0.705	7.1
Wang et al. Int J Cardiovasc Imaging 2020 [14]	121	0.02	(-0.16, 0.2)	0.847		1.30	(-11.92, 14.52)	0.847	8.7
Lin et al. J Magn Reson Imaging 2017 [15]	50	-0.91	(-1.24, -0.58)	0.000		-14.80	(-19.32, -10.28)	0.000	6.9
Vincenti et al. JACC Cardiovasc Imaging 2014 [16]	33	-0.97	(-1.38, -0.56)	0.000		-9.90	(-13.38, -6.42)	0.000	5.9
Pooled (random effects) model		-0.15	(-0.32, 0.01)	0.059		-2.39	(-4.7, -0.07)	0.043	

Differences in LVEDV between compressed-sensing and control sequences are presented as standardized difference and mean difference with effects pooled using a random effects model

**Table 3:** Study-specific differences in left ventricular end-systolic volume

Study name	Sample Size	Standardized difference	95% CL	p value		Mean difference, mL	95% CL	p value	Weight
Allen, et al. Int J Cardiovasc Imaging 2016 [2]	29	0.09	(-0.65, 0.83)	0.816		2.70	(-20.08, 25.48)	0.816	2.6
Ma et al. Clinical Radiology 2019 [3]	33	0.24	(-0.11, 0.58)	0.178		0.39	(-0.17, 0.95)	0.172	6.8
Kido et al. JCMR 2021 [4]	65	0.05	(-0.2, 0.29)	0.706		0.30	(-1.26, 1.86)	0.705	8.7
Kido et al. JCMR 2016 [5]	81	-0.01	(-0.44, 0.43)	0.982		-0.20	(-17.71, 17.31)	0.982	5.3
Goebel et al. JMRI 2016 [6]	16	0.08	(-0.41, 0.57)	0.743		1.30	(-6.47, 9.07)	0.743	4.7
Goebel et al. Eur Radiology 2016 [7]	26	0.11	(-0.66, 0.88)	0.776		3.00	(-17.6, 23.6)	0.775	2.4
Goebel et al. Acta Radiology 2017 [8]	20	0.62	(0.14, 1.1)	0.011		8.60	(2.56, 14.64)	0.005	4.8
Allen, et al. Eur Radiology 2018 [9]	27	-0.41	(-1.19, 0.37)	0.299		-25.40	(-72.82, 22.02)	0.294	2.4
Sudarski et al. Radiology 2016, [10] Patients	50	-0.08	(-0.36, 0.2)	0.577		-0.80	(-3.61, 2.01)	0.577	8.0
Sudarski et al. Radiology 2016, [10] Ctrl subjects	10	0.28	(-0.35, 0.92)	0.377		2.90	(-3.41, 9.21)	0.368	3.3
Naresh et al. Pediatric Radiology 2020 [11]	28	-0.09	(-0.46, 0.28)	0.642		-3.00	(-15.61, 9.61)	0.641	6.3
Kocaoglu et al. J Cardiovasc Magn Reson 2020 [12]	26	-0.27	(-0.66, 0.12)	0.172		-1.20	(-2.89, 0.49)	0.164	6.0
Wang et al. SS CS Cardiovasc Diagn Ther 2020 [13]	38	0.07	(-0.24, 0.39)	0.649		0.90	(-2.97, 4.77)	0.648	7.2
Wang et al. 2-shot SS Cardiovasc Diagn Ther 2020 [13]	38	0.01	(-0.31, 0.33)	0.955		0.10	(-3.38, 3.58)	0.955	7.3
Wang et al. Int J Cardiovasc Imaging 2020 [14]	121	0.02	(-0.16, 0.2)	0.832		0.80	(-6.57, 8.17)	0.832	10.0
Lin et al. J Magn Reson Imaging 2017 [15]	50	-0.74	(-1.06, -0.43)	0.000		-8.40	(-11.53, -5.27)	0.000	7.3
Vincenti et al. JACC Cardiovasc Imaging 2014 [16]	33	-0.17	(-0.51, 0.17)	0.330		-2.00	(-5.99, 1.99)	0.326	6.8
Pooled (random effects) model		-0.03	(-0.16, 0.11)	0.679		-0.47	(-1.89, 0.95)	0.519	

Differences in LVESV between compressed-sensing and control sequences are presented as standardized difference and mean difference with effects pooled using a random effects model

**Table 4:** Study-specific differences in left ventricular mass

Study name	Sample Size	Standardized difference	95% CL	p value		Mean difference, g	95% CL	p value	Weight
Allen, et al. Int J Cardiovasc Imaging 2016 [2]	29	0.14	(-0.6, 0.88)	0.712		5.20	(-22.39, 32.79)	0.712	3.9
Ma et al. Clinical Radiology 2019 [3]	33	-0.12	(-0.46, 0.22)	0.495		-0.51	(-1.97, 0.95)	0.494	8.0
Kido et al. JCMR 2021 [4]	65	-0.15	(-0.4, 0.09)	0.221		-0.90	(-2.33, 0.53)	0.219	9.3
Kido et al. JCMR 2016 [5]	81	-0.04	(-0.48, 0.39)	0.845		-1.20	(-13.23, 10.83)	0.845	6.8
Goebel et al. JMRI 2016 [6]	16	0.27	(-0.23, 0.77)	0.287		9.30	(-7.53, 26.13)	0.279	6.0
Goebel et al. Eur Radiology 2016 [7]	26	0.06	(-0.71, 0.83)	0.877		2.00	(-23.33, 27.33)	0.877	3.7
Goebel et al. Acta Radiology 2017 [8]	20	0.22	(-0.22, 0.67)	0.323		2.70	(-2.59, 7.99)	0.317	6.7
Allen, et al. Eur Radiology 2018 [9]	27	0.21	(-0.56, 0.99)	0.585		6.00	(-15.47, 27.47)	0.584	3.7
Sudarski et al. Radiology 2016, [10] Patients	50	0.89	(0.56, 1.22)	0.000		8.70	(5.99, 11.41)	0.000	8.2
Sudarski et al. Radiology 2016, [10] Ctrl subjects	10	1.39	(0.52, 2.26)	0.002		10.40	(5.75, 15.05)	0.000	3.1
Kocaoglu et al. J Cardiovasc Magn Reson 2020 [12]	26	0.47	(0.06, 0.87)	0.024		2.00	(0.35, 3.65)	0.018	7.2
Wang et al. SS CS Cardiovasc Diagn Ther 2020 [13]	38	0.04	(-0.28, 0.36)	0.818		0.90	(-6.74, 8.54)	0.817	8.3
Wang et al. 2-shot SS Cardiovasc Diagn Ther 2020 [13]	38	0.05	(-0.27, 0.36)	0.778		0.80	(-4.76, 6.36)	0.778	8.3
Lin et al. J Magn Reson Imaging 2017 [15]	50	0.08	(-0.2, 0.35)	0.590		0.90	(-2.37, 4.17)	0.590	8.9
Vincenti et al. JACC Cardiovasc Imaging 2014 [16]	33	0.26	(-0.09, 0.61)	0.141		2.50	(-0.78, 5.78)	0.135	7.9
Pooled (random effects) model		0.20	(0.02, 0.38)	0.026		2.65	(0.57, 4.73)	0.012	

Differences in LVM between compressed-sensing and control sequences are presented as standardized difference and mean difference with effects pooled using a random effects model

**Table 5:** Study-specific differences in left ventricular stroke volume

Study name	Sample Size	Standardized difference	95% CL	p value		Mean difference, mL	95% CL	p value	Weight
Allen, et al. Int J Cardiovasc Imaging 2016 [2]	29	0.16	(-0.59, 0.9)	0.681		4.80	(-18.05, 27.65)	0.681	3.9
Ma et al. Clinical Radiology 2019 [3]	33	-0.20	(-0.55, 0.14)	0.246		-0.62	(-1.66, 0.42)	0.241	10.5
Kido et al. JCMR 2021 [4]	65	-0.06	(-0.3, 0.18)	0.622		-0.60	(-2.98, 1.78)	0.622	13.8
Kido et al. JCMR 2016 [5]	81	-0.06	(-0.5, 0.38)	0.796		-1.00	(-8.58, 6.58)	0.796	8.2
Goebel et al. Eur Radiology 2016 [7]	26	-0.45	(-1.23, 0.33)	0.257		-10.00	(-27.09, 7.09)	0.251	3.6
Goebel et al. Acta Radiology 2017 [8]	20	-0.16	(-0.6, 0.28)	0.486		-2.90	(-11, 5.2)	0.483	8.1
Allen, et al. Eur Radiology 2018 [9]	27	-0.10	(-0.87, 0.67)	0.791		-2.20	(-18.5, 14.1)	0.791	3.7
Sudarski et al. Radiology 2016, [10] Patients	50	-0.26	(-0.54, 0.02)	0.074		-2.30	(-4.78, 0.18)	0.069	12.5
Sudarski et al. Radiology 2016, [10] Ctrl subjects	10	0.01	(-0.61, 0.63)	0.967		0.10	(-4.66, 4.86)	0.967	5.2
Kocaoglu et al. J Cardiovasc Magn Reson 2020 [12]	26	-0.16	(-0.54, 0.23)	0.432		-0.90	(-3.13, 1.33)	0.429	9.4
Lin et al. J Magn Reson Imaging 2017 [15]	50	-0.75	(-1.07, -0.44)	0.000		-6.40	(-8.76, -4.04)	0.000	11.5
Vincenti et al. JACC Cardiovasc Imaging 2014 [16]	33	-0.68	(-1.06, -0.3)	0.000		-8.70	(-13.07, -4.33)	0.000	9.6
Pooled (random effects) model		-0.26	(-0.42, -0.1)	0.002		-2.52	(-4.31, -0.73)	0.006	

Differences in LVSV between compressed-sensing and control sequences are presented as standardized difference and mean difference with effects pooled using a random effects model

### Analysis of acceleration factor

**Fig. 5:**
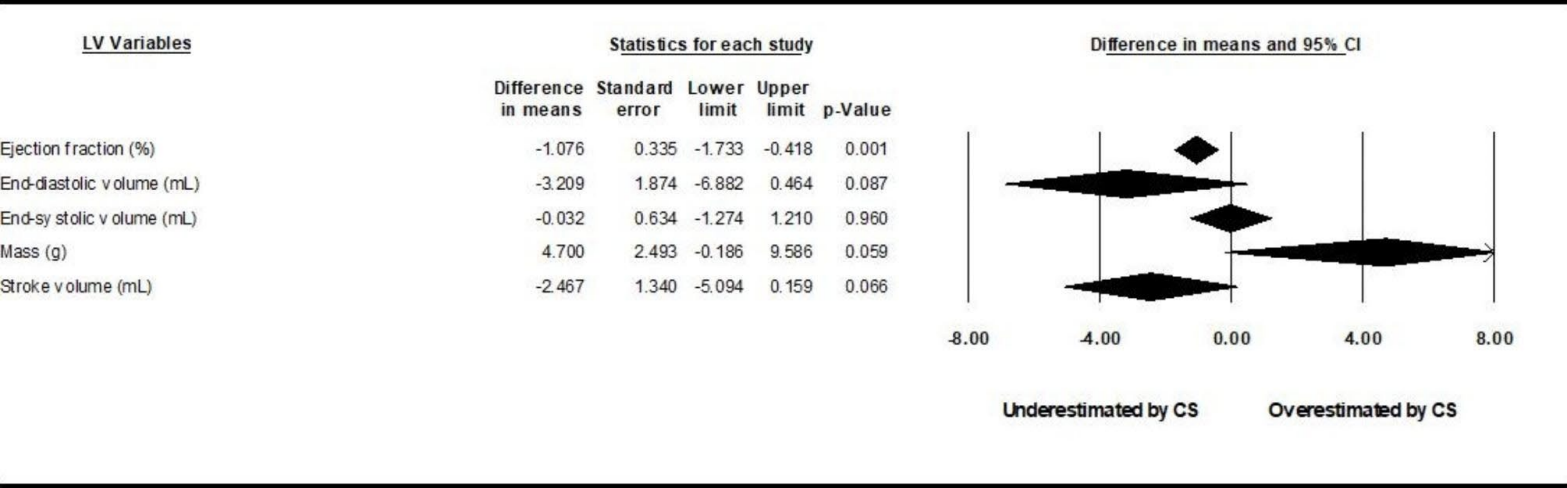
**Subgroup analysis results, high acceleration factor (≥ 11).** Comparison of sequences stratified by acceleration factor. High (≥ 11) acceleration factor CS sequences demonstrated non-significant differences except slightly underestimated LVEF

**Fig. 6:**
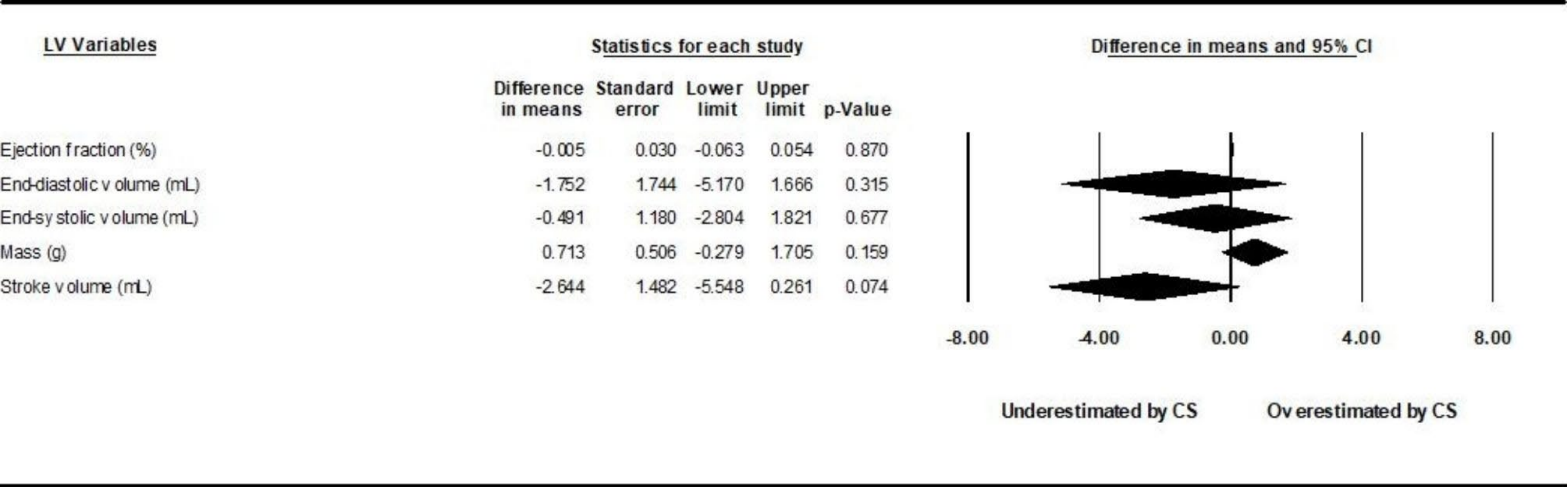
**Subgroup analysis results, low acceleration factor (< 11).** Low (< 11) acceleration factor sequences demonstrated attenuated non-significant differences compared with all pooled studies

Finally, to study the effect of acceleration rate on quantitative parameters, the 12 Siemens CS studies were divided into 4 studies with exclusively low (< 11) acceleration factors, and six studies with exclusively high (≥ 11) acceleration factors. One study used both high and low acceleration factors; the remaining study did not state an overall acceleration factor (could not be determined). The results are provided in Figs. 5 and 6. There were no statistically significant differences in LVM, LVEDV, LVSV, LVESV, or observed compared to the gold standard. LVEF measured in high acceleration factor studies was slightly underestimated (1.08%, p = 0.001) by CS cine sequences.

## Discussion

### Development of compressed sensing and parallel imaging

Parallel imaging was developed in the late 1990s after the availability of radiofrequency coil arrays [21]. This method can accelerate segmented cine CMR by undersampling k-space. A special image reconstruction algorithm is used which can either suppress image-space aliasing artifacts caused by k-space undersampling or recover missing k-space data directly. Two representative parallel imaging techniques are SENSE [22] and GRAPPA [23]. SENSE is an image-space reconstruction technique that can suppress aliasing artifacts by directly using multi-channel coil sensitivity encoding. GRAPPA is a k-space technique that can implicitly utilize multi-channel coil sensitivity encoding to calibrate k-space data relationship for data recovery. Both SENSE and GRAPPA use cartesian sampling with a linear reconstruction algorithm, providing a practically effective and computationally affordable method for segmented cine. Because segmented cine data are collected dynamically, they can benefit from time-domain data correlation. This enables a set of k-t space acceleration techniques, including k-t GRAPPA [24] and k-t SENSE/BLAST [25], for further imaging acceleration.

CS reconstruction was introduced for MRI applications more recently in the 2000s [26]. This method relies on data sparsity naturally existing in medical images. A random sampling strategy is required for generating incoherence of undersampling artifacts in image space. By non-linearly enforcing both image sparsity and data consistency with the acquired samples, the image artifacts can be effectively suppressed. Because cardiac images have high sparsity in k-t space, CS found applications rapidly in the field of CMR. CS cine was developed to overcome two challenges in conventional segmented cine: first, breath holding is difficult in many heart patients; second, ECG synchronization for data segmentation may not be effective for arrhythmia patients. Ideally, CS cine would accelerate CMR beyond parallel imaging that is limited by MRI coil array, thereby allowing for real-time cine with free breathing. However, CS cine may lower image resolution because sparsity enforcement may smooth the images either in image space or along the time. Because CS requires random sampling, it is suitable for non-Cartesian sampling trajectories that naturally produce noise-like (incoherent) aliasing artifacts. However, non-Cartesian sampling may suffer from k-space trajectory inaccuracy due to gradient imperfection, manifesting as image blurs or distortion. For this reason, Cartesian sampling is preferred in clinical applications. Cartesian CS typically uses pseudo-random sampling in k-space [27–29]. The reconstruction algorithm is non-linear and iterative, thereby requiring more reconstruction time than parallel imaging. A high-performance computer is usually needed for CS reconstruction in a clinical environment.

### Iterative SENSE and CS SENSE

Many research studies have combined parallel imaging and CS together [30, 31], making it possible to take advantage of both multi-channel coil sensitivity encoding and image sparsity in imaging acceleration. To that end, iterative SENSE has been found to be useful because it features an iterative algorithm and an arbitrary sampling trajectory [32] that are both needed for the application of CS sparsity constraint. In the presented work, all the 15 studies relied on the CS combined with iterative SENSE. The Cartesian data were sampled with higher density around the central k-space than that in the peripheral k-space. This variable density sampling allowed for image reconstruction without reference scans like in SENSE and thus improved overall imaging acceleration. It was reported that the combination of CS and SENSE provided high acceleration factor (typically ≥ 8) without considerable loss in image resolution. However, these CMR cine prototypes required significant computation in image reconstruction. To reduce reconstruction time, the algorithm is now implemented practically with graphic processing unit (GPU) [33]. This has fulfilled clinical needs in most cases with a higher cost on computer hardware.

### Overview: reproducibility of measurements

To our knowledge, this is the first meta-analysis comparing CS cine with the gold standard bSSFP segmented cine. Several factors can influence the reproducibility of left and right ventricular volumetric measurements. Miller et al. showed the ideal spatial resolution for cardiac cine was < 2 mm, with a temporal resolution ≤ 45 ms [34]. With decreasing temporal resolution (reduction in the number of true cine frames) on segmented sequences, true end systole could be missed, leading to an overestimation of LVESV and underestimation of LVEF. Decreasing spatial resolution was associated with increased LVEDV. True voxel size up to 3 mm and true cine frame intervals up to 90 ms were not shown to affect LVM. All included studies met the former specification for acceptable spatial and temporal resolution; however, several studies have noted significant differences in left ventricular volumetric data as well as LVM compared to the reference. These differences included underestimation as well as overestimation of LVEDV, LVESV, LVSV and LVM. The net effect of the meta-analysis has shown overall, although statistically significant differences in LVSV, LVEDV, and LVM exist, they are unlikely to be clinically impactful.

### Effect of gating method

Because of the inherent trigger delay with prospective gating, true end-diastole is not captured, resulting in smaller end diastolic volumes, stroke volumes, and ejection fractions [35]. Alternatively, retrospective and real-time imaging also became feasible without the use of offline reconstruction. Ungated sequences could be of particular value at higher field strengths given the burden of magnetohydrodynamic effects on EKG T-wave amplitude.

Differences in LVM persisted across several studies with different gating methods. Individual author observations included the following: Sudarski et al. identified overestimation of LVM with prospective gating. Kocaoglu et al. noted overestimation of LVM using breath hold CS cine vs. reference, but no significant difference in free breathing CS cine vs. reference. After indexing for BSA, there was no significant difference between breath hold CS cine and reference. Kido et al. [JCMR, 2016] noted underestimation of LVM with CS cine using prospective gating covering > 1 cardiac cycle. Ma et al. noted underestimation in LVM with CS cine using retrospective gating. In several studies, CS cine showed increased or decreased LVEDV, LVSV, LVEF, and increased LVESV regardless of gating method used. In our subgroup analyses however, prospectively gated CS cine sequence studies demonstrated no significant differences between LV volumes and mass except for LVSV which was slightly underestimated.

### Image contrast, spatial-temporal blurring, and post-processing

#### Acceleration factors

CS substantially improves quality of real-time imaging, which may be comparable to the reference based on several of the included studies. Increased spatial-temporal undersampling occurs at higher acceleration factors. As mentioned, tradeoffs for consideration include decreased image contrast, temporal blurring, and decreased image sharpness which is linked to the degree of undersampling. Alternatively, CS cine with segmented imaging can be used with a substantially shortened breath hold duration using a smaller acceleration factor. The studies in this meta-analysis used an acceleration factor ranging from 2.5 to 4 for the CS cine prototypes on the Philips MRI scanners, and 4-12.8 for the CS cine prototypes on the Siemens MRI scanners. In the group of Siemens CS cine prototypes, differences in LV volumes/and or mass occurred in studies with acceleration rates of > = 11 (high) regardless of gating method (total 6 of 12 studies). 4 out of 12 studies exclusively used acceleration rates < 11 (low) and showed no difference compared to reference[2, 13, 14]. Only one study from the high acceleration pool showed no significant difference vs. reference[5]. One study contained high and low acceleration rates and showed no difference[9]; one study only included differential subsampling rates so the overall acceleration factor could not be determined[6]. In the group of Philips CS cine prototypes, only 3 studies were included that used the CS-SENSE method[3, 11, 12]. Statistically significant differences in left ventricular quantitative parameters were present in 2 studies using the acceleration rates of 3 and 4 compared to 2.5-3.5x acceleration. Due to the relatively small number of studies on the Philips MRI scanners, subgroup analysis based on acceleration rate from this vendor could not be performed. Overall, stratified subgroup-analysis based on acceleration factor could not explain the differences in the non-stratified comparison, and only a trivial underestimation in LVEF among the high acceleration group was appreciated. Low overall mean differences in stroke volume, LVEDV, and LVM in the aggregate analysis likely influenced the result of this particular subgroup analysis.

#### Left ventricular papillary muscles and trabeculations

Another factor affecting left ventricular quantitative measurements in clinical practice is inclusion/exclusion of the papillary muscle into the left ventricular volume or mass. Only one study definitively excluded papillary muscles from the left ventricular volume. Han et al. demonstrated papillary muscle and trabeculation inclusion resulted in a 17% higher indexed LVM, 20% lower indexed LVEDV, and 13% higher LVEF in patients with hypertrophic cardiomyopathy. Imaging at a higher spatial resolution may be necessary to successfully depict small left ventricular trabeculation and myocardial crypts if inclusion into the left ventricular mass is desired [36]. Additionally, thresholding techniques may be affected by decreased image contrast [37], and performing left ventricular cine after infusion of gadolinium based contrast agents (GBCA). These considerations may be important when using higher acceleration factors.

#### Potential implications of readout: GRE vs. bSSFP

The T2/T1 weighting of bSSFP and high flip angles result in a darker appearance of the myocardium compared to gradient recall echo (GRE). The flow sensitivity of GRE also affects image contrast and visualization at the sub-endocardial/blood pool interface. These factors lead to differences in volumetric measurements compared to bSSFP [38]. Due to the increased utilization of MRI in patients with MR conditional and non-conditional devices and use of higher field strengths, CS cine with GRE readout would meet a growing unmet clinical need in this population. However, decreases in myocardial-blood pool CNR with higher acceleration factors may be more adversely impactful when combined with GRE readout.

### The effect of physiologic variation

Inter-study physiologic variation must also be considered, which is particularly relevant when comparing breath hold techniques with free breathing techniques. Although image quality has been demonstrated to be adequate under these circumstances, the free breathing acquisition may introduce respiratory dependent differences in left and right ventricular volumes, introduce through plane cardiac motion, and non-matched slice positions compared to end-expiratory breath holds. On the other hand, free breathing CS offers better spatial and temporal resolution compared to conventional parallel imaging; thus, the impact of pathophysiology on ventricular filling dynamics, (i.e. interventricular dependence) can be studied with free breathing (with potentially greater sensitivity).

### Barriers to CS cine adoption

As productivity and economic based concerns and incentives continue to grow and the computational processing power floor is raised, net benefit measures may favor more widespread adoption of CS cine. Some barriers to this adoption still exist, but are being addressed.

More limited data regarding the suitability of CS cine exist in the pediatric population, where there is more demand on temporal resolution, and acquired voxel size. Pediatric patients were aggregated and overall represent a small sample of patients in the included studies (2 included studies)[11, 12]. More recent studies subsequent to the search scope of this meta-analysis appear to favorably support the use of CS in this population [39]; however, some differences in LVEF and right ventricular EF compared to the reference have been appreciated [40]. Using our methodology for inclusion/exclusion, the majority of studies did not include right ventricular quantitative data (only 2 studies were representative); therefore, differences in right ventricular quantitative measurements obtained from CS cine could not be compared with the gold standard. Only one study included left ventricular strain as a variable, which was global circumferential strain[4]. Further comparisons are needed with the addition of other strain parameters such region, global longitudinal and global radial strain.

### Summary of recommendations

The findings of the meta-analysis suggests that CS cine can be used for assessment of left ventricular function and volumes in certain populations. The included studies in this meta-analysis consisted mostly of adult patients. It is reasonable to conclude that longitudinal assessment with serial CMR is feasible between conventional parallel imaging and CS. Tradeoffs for consideration include patient acuity, R-R interval irregularity, need to resolve fine details that are spatial-temporal dependent (i.e. cardiac valves), post-processing technique, preservation of image contrast, workflow throughput, and evaluation of the right ventricle.

### Limitations

In addition to what has already been discussed, there are some additional limitations to the present study, namely study heterogeneity. Acquisition parameters and technique varied across the studies included in the meta-analysis. This not only included spatial and temporal resolution, which have an impact on quantitative measurements, but also free breathing, breath hold, multi-shot, and single shot acquisition strategies. Given the limited number of included studies, further subgroup analysis addressing these differences could not be performed. Within the subgroup analysis performed (gating method and acceleration factor), confidence intervals were generally wider, which was due to dividing the number of studies included in the aggregate analysis. This is particularly true of the acceleration factor subgroup analysis, with a lower number of included studies compared to the gating subgroup analysis. We also acknowledge our limited ability to detect possible publication bias due to the small number of studies. Lastly, CS methods have been applied to 3D cine which can provide comparable left ventricular functional and morphological assessment [41]. The current meta-analysis only examined CS application to 2D cine.

## Conclusions

CS cine provides accurate assessment of left ventricular structure and function when compared to conventional cine imaging. Small differences are observed overall in LVEDV, LVSV, and LVM.

### Electronic supplementary material

Below is the link to the electronic supplementary material.

Supplementary Material 1

## Data Availability

Datasets analyzed for the current study were obtained from the source publications (references 2–16). However, these can be provided upon reasonable request from the corresponding author.

## List of Abbreviations

BLAST: Broad-use linear acquisition speed-up technique
BSA: Body surface area
bSSFP: Balance steady-state free precession
CMR: Cardiac MRI
CNR: Contrast to noise
CS: Compressed sensing
C-SENSE: Compressed SENSE
EKG: Electrocardiogram
GBCA: Gadolinium based contrast agent
GRAPPA: Generalized autocalibrating partial parallel acquisition
GRE: Gradient recall echo
LVEDV: Left ventricular end diastolic volume
LVEF: Left ventricular ejection fraction
LVESV: Left ventricular end systolic volume
LVM: Left ventricular mass
LVSV: Left ventricular stroke volume
PRISMA: Preferred reporting items for systematic reviews and meta-analyses
SENSE: Sensitivity encoding
