# Supplementary material for: Comparison between compressed sensing and segmented cine cardiac magnetic resonance: a meta-analysis
Source: BMC Cardiovasc Disord. 2023 Sep 21;23:473. doi: 10.1186/s12872-023-03426-1 (PMC10512640; doi:10.1186/s12872-023-03426-1)
Supplement: Supplementary file 1 — Supplementary Material 1 [file 12872_2023_3426_MOESM1_ESM.docx]

ONLINE SUPPLEMENT

Title: Comparison between compressed sensing and segmented cine cardiac magnetic resonance: A meta-analysis

Authors: Jason Craft, MD^1,^, Yulee Li, PhD^1^, Niloofar Fouladi Nashta, MPH^2^, Jonathan Weber, MPH^1^

^1^DeMatteis Cardiovascular Institute, St. Francis Hospital & Heart Center, Roslyn, NY

^2^Sol Price School of Public Policy and Leonard D. Schaeffer Center for Health Policy and Economics, University of Southern California, Los Angeles, CA, USA

Author emails:

Jason Craft: [Jason.Craft@chsli.org](mailto:Jason.Craft@chsli.org)

Yulee Li: [Yulee.li@chsli.org](mailto:Yulee.li@chsli.org)

Niloofar Fouladi Nashta: [niloofar.fouladi@gmail.com](mailto:niloofar.fouladi@gmail.com)

Jonathan Weber: [Jonathan.Weber@chsli.org](mailto:Jonathan.Weber@chsli.org)

Corresponding author:

Jason Craft, MD

St. Francis Hospital & Heart Center

100 Port Washington Blvd.

Roslyn, NY 11576

516 622-4552

Table S1. Included studies, acquisitions techniques, and post-processing

| **Author** | **Journal and Year** | **Sample**  **Size** | **Inline reconstruction method** | **CS Gating method** | **Papillary muscle in volume** | **Breath hold** | **Acceleration factor** |
| --- | --- | --- | --- | --- | --- | --- | --- |
| Allen et al. [2] | Int J Cardiovasc Imaging 2016 | 29 | Siemens CS-SENSE: Iterative SENSE with CS (wavelet) constraint along time and space (phase encoding). | prospective  (segmented) | excluded | breath hold | 4 |
| Ma et al. [3] | Clinical Radiology 2019 | 33 | Philips CS-SENSE: Unknown details about image reconstruction. | retrospective  (segmented) | included | breath hold and free breathing | 4 |
| Kido et al. [4] | JCMR 2021 | 65 | Siemens CS-SENSE: Iterative SENSE with CS (wavelet) constraint along time and space (phase encoding). | prospective >1 cardiac cycle  (single-shot) | unclear | breath hold | 12.8 |
| Kido et al. [5] | JCMR 2016 | 81 | Siemens CS-SENSE: Iterative SENSE with CS (wavelet) constraint along time and space (phase encoding). | prospective >1 cardiac cycle  (single-shot) | included | breath hold | 12.8 |
| Goebel et al. [6] | JMRI 2016 | 16 | SPARSE-SENSE: Research version, Combined SENSE and CS. | prospective  (segmented) | included | multibreath-hold (1 per short axis slice) | high spatial resolution/ central k-space:3 and peripheral k-space:14; high temporal resolution/ central k-space: 1 and peripheral k-space: 17 |
| Goebel et al. [7] | Eur Radiology 2016 | 26 | SPARSE-SENSE: Research version, Combined SENSE and CS | real-time  (ungated) | unknown | free breathing | overall 11, 5 (central k-space), 13 (peripheral k-space) |
| Goebel et al .[8] | Acta Radiology 2017 | 20 | SPARSE-SENSE: Research version, Combined SENSE and CS | real-time  (ungated) | unclear | free breathing | 5 (central k-space), 13 (peripheral k-space) |
| Allen, et al. [9] | Eur Radiology 2018 | 27 | Siemens CS-SENSE: Iterative SENSE with CS (wavelet) constraint along time and space (phase encoding). | real-time  (ungated) | unclear | breath hold | 9.9-12 |
| Sudarski et al. [10] | Radiology 2016 | 10 normals, 50 patients | Siemens CS-SENSE: Iterative SENSE with CS (wavelet) constraint along time and space (phase encoding). | prospective  (single-shot) | included | free breathing and breath hold | 11.3 |
| Naresh et al. [11] | Pediatr Radiology 2021 | 28 | Philips CS-SENSE: Unknown details about image reconstruction. | retrospective  (segmented) | unclear | breath hold | 2.5-3.5 |
| Kocaoglu et al. [12] | J Cardiovasc Magn Reson 2020 | 26 | Philips CS-SENSE: Unknown details about image reconstruction. | retrospective  (segmented) | included | free breathing and breath hold | 3 |
| Wang et al. [13] | Cardiovasc Diagn Ther 2020 | 38, counted twice, 2 sequences | Siemens CS-SENSE: Iterative SENSE with CS (wavelet) constraint along time and space (phase encoding). | adaptive prospective  (single-shot), retrospective (segmented) | included | breath hold | 6.5-9.5 |
| Wang et al. [14] | Int J Cardiovasc Imaging 2020 | 121 | Siemens CS-SENSE: Iterative SENSE with CS (wavelet) constraint along time and space (phase encoding). | unknown | included | breath hold | 9.9 |
| Lin et al. [15] | J Magn Reson Imaging 2017 | 50 | Siemens CS-SENSE: Iterative SENSE with CS (wavelet) constraint along time and space (phase encoding). | prospective <1 cardiac cycle  (segmented) | included | breath hold | 8 |
| Vincenti et al. [16] | JACC Cardiovasc Imaging 2014 | 33 | Siemens CS-SENSE: Iterative SENSE with CS (wavelet) constraint along time and space (phase encoding). | prospective <1 cardiac cycle  (segmented) | included | breath hold | 11 |

Legend: There were 15 studies which met inclusion criteria for our meta-analysis. Sample size ranged from 16-121 participants across 2 vendors, 1.5 T and 3T, with comparison against segmented or real-time acquisition control sequence.

Table S2. Sequence parameters, measurements collected, and study findings

| **Author** | **Field strength** | **Spatial and temporal resolution (reference)** | **Spatial and temporal resolution (CS)** | **Data variables** | **Findings** | **Scan reduction time** |
| --- | --- | --- | --- | --- | --- | --- |
| Allen et al. [2] | 1.5T | 1.9 X 1.9 mm, TRES 40.8 ms | 2 X 2 mm, TRES 38.1 ms | LVEF, LVEDV, LVESV, LVSV, LVCO LVM, Image quality | No significant difference in quantitative cardiac function was present between the three techniques. There was a non-significant trend towards higher end-diastolic volume in the IS SENSE acquisition. Image quality was equivalent between the reference GRAPPA technique and IS SENSE, and both superior to TSENSE. | 64% reduction in breath hold duration |
| Ma et al. [3] | 3T | 1.7 X 1.7 mm. TRES 41 ms | 1.2 X 1.2 mm, TRES 41ms for both CS cine breath hold and free breathing | LVEDV, LVESV, LVSV,LVEF, LVM, regional myocardial wall motion | There were no significant differences in LVEDV, LVESV, LVSV, or LV EF measurements among the three datasets. Relative to the reference standard, however, a small but significant underestimation of LVED mass was noted with the csBTFE-FB sequences. LVED mass and regional myocardial wall motion were underestimated on csBTFE-FB and csBTFE-BH. | > 70% reduction in scan time |
| Kido et al. [4] | 3T | 1.7 X 1.7 mm, TRES 41.1 ms | 1.7 X 1.7 mm, TRES 41.1 ms | LVEDV, LVESV, LVSV, LVM, LVEF | No significant difference in LVEDV, ESV, SV, but free breathing CS underestimated LV mass | 79% scan time reduction |
| Kido et al. [5] | 3T | 1.7 X 1.7 mm, TRES 41 ms | 1.7 X 1.7 mm, TRES 41 ms | LVEDV, LVESV, LVSV, LVM, LVEF, GCS, GCS rate | There were no statistically significant differences in the GCS and GCS rate. No significant differences were found in all LV volume assessment between both types of cine CMR. | 78% scan time reduction |
| Goebel et al. [6] | 1.5 T | 1.7 X 1.7 mm, TRES 40 ms | high spatial resolution: 1.0 X 1.0 mm, TRES 40 ms; high temporal resolution: 1.7 X 1.7 mm, TRES 11 ms | LVEDV, LVESV, LVSV, LVEF, LVM | LVSV, LVEF and LVM had small but significant decreases observed in the high spatial resolution group. No difference in LVEDV and LVESV. LVESV and LVM had statistically significant decreases in the high temporal resolution group. No significant difference in LVEDV, LVSV, LVEF. | unknown |
| Goebel et al. [7] | 1.5 T | 1.7 X 1.7 mm, TRES 38 ms | 1.8 X 1.8 mm, TRES 39 ms | LVEDV, LVESV, LVSV, LVEF, LVM | Statistically significant minor decreases for LVEDV, LVEF, LVSV, but no difference for LVM with CS. Significant increase in LVESV. | 92% scan time reduction |
| Goebel et al. [8] | 1.5 T | 1.8 X 1.8 mm, TRES 45.9 ms | 1.8 X 1.8 mm, TERES 39 ms | LVEDV, LVESV, LVSV, LVEF, LVM | Small, but significantly significant increase in LVESV for CS cine, but no statistically significant difference seen for LVEDV, LVSV, LVEF, LVM. CS had significantly less arrhythmia-caused artifacts in comparison to reference SSFP images. | 97% scan time reduction |
| Allen, et al. [9] | 1.5 T | 1.8 X 1.8 mm TRES 42 ms | 2 X 2 mm, TRES 42 ms | LVEDV, LVESV, LVEF, LVSV, LVCO, LVM | No statistical difference in EF, EDV, ESV, SV, CO, or myocardial mass. IS SENSE acceleration has improved overall image quality in patients with atrial fibrillation and generally equivalent qualitative performance in patients in sinus rhythm relative to segmented acquisitions. | unknown |
| Sudarski et al. [10] | 3T | 1.5 X 1.5 mm, Tres 43 | 2 X 2 mm, TRES 34 ms | LVEDV, LVESV, SV, LVM, LVEF | Quantification of LV function similar for CS versus reference irrespective of breath hold commands. There is overestimation of LVM with CS irrespective of breath holding. | 90-93% time reduction |
| Naresh et al. [11] | 3T | .8 -1.4 mm X .8-1.4 mm, TRES 17-38 ms | 0.8-1.4 mm X .8-1.4 mm, TRES 17-42 ms | LVEDV, LVESV, LVEF, LVCO, RVEDV, RVESV, RVEF, RVCO | LVESV and RVEF approached significant difference | 43% scan time reduction |
| Kocaoglu et al. [12] | 1.5 T | 1.6-1.7 mm X 1.6-1.7 mm, TRES 40-45 ms | 1.6-1.7 mm X 1.6-1.7 mm, TRES 40-45 ms | LVEDV, LVESV, LVEDVI, LVSV, LVSVI, LVEF, LVM, LVMI, RVEDV, RVESV, RVEF, RVEDVI, RVESVI, RVSV, RVSVI, RVEF | Statistically significant increase in LVM; decrease in RVEDV, RVEDVi, RVESV, RVESVI between reference segmented and breath hold CS cine. Free breathing CS had higher LVEDV, LVEDVI and higher LVESV compared to reference. | breath hold duration reduction of 33% |
| Wang et al. [13] | 3T | 1.6 X 1.6 mm, TRES 45 ms | 1.6-1.7 mm X 1.5-1.7 mm TRES 42 ms | LVEDV, LVESV, LVEF, LVCO, Lvmass | Significant difference in image quality with single shot CS compared with reference segmented. No difference between 2 shot CS and reference segmented. | 70-80% scan time reduction |
| Wang et al. [14] | 3T | 1.7 X 1.7 mm, TRES 45 ms | 1.8 X 1.8 mm, TRES 44 ms | LVEDV, LVESV, LVMI, LVEF | No significant difference between CS and reference segmented cine | 88% scan time reduction |
| Lin et al. [15] | 1.5 T | 1.25 X 1.25 mm TRES 30 ms | 1.5 X 1.5 mm TRES 30 ms | LVEDV, LVESV, LVSV, LVEF, LVM | No significant differences in volumetrics. No significant distance in regional wall motion. | 80% scan time reduction |
| Vincenti et al. [16] | 1.5 T | 1.2 X 1.2 mm TRES 49 m | 1.5 X 1.5 mm TRES 30 ms | LVEF, LVEDV, LVESV, LVSV, LVmass | LVEDV significantly higher with reference segmented cine | 76% scan time reduction |

Legend: Each of the 15 studies included in meta-analysis included LVESV, LVEDV, and LVEF. Most studies also included LVM and LVSV. Reported scan reduction time or breath-hold reduction time ranged from 33-97%.

Table S3. Evaluation of publication bias

| **Parameter** | **Original** | **Trim-and-fill corrected** | **Kendall's Tau** | | **Egger's test** |
| --- | --- | --- | --- | --- | --- |
|  | *Standardized difference* | *Standardized difference* | *Tau* | *p-value* | *p-value* |
| **LVEDV** | -0.15473 (-0.31511, 0.00565) | -0.22030 (-0.36596, -0.07464) | -0.021 | 0.25 | 0.59 |
| **LVESV** | -0.02841 (-0.16305, 0.10624) | -0.09386 (-0.23211, 0.04439) | 0.03 | 0.87 | 0.77 |
| **LVEF** | -0.08795 (-0.17085, -0.00505) | -0.08456 (-0.16699, -0.00213) | -0.12 | 0.51 | 0.5 |
| **LV Mass** | 0.20409 (0.02411, 0.38408) | 0.33937 (0.15159, 0.52716) | 0.29 | 0.14 | 0.2 |
| **LV Stroke Volume** | -0.25916 (-0.42297, -0.09536) | -0.29246 (-0.44895, -0.13596) | 0.09 | 0.68 | 0.68 |

Legend: Publication bias was evaluated through the use of the trim-and-fill method applied to funnel plots as well as Kendall’s Tau and Egger’s Test for interdependence of variance and effect size, and asymmetry of the funnel plots, respectively. Findings demonstrate a lack of both significant change in standardized differences, interdependence, and asymmetry. Publication bias findings are limited and may be underestimated due to the small number of studies evaluated.
